# A Standardized Definition of Rapid Evidence Assessment for Environmental Applications

**DOI:** 10.1111/con4.70005

**Published:** 2026-01-14

**Authors:** J. Angus Webb, Kate A. Schofield, Carly N. Cook, Jonathan R. B. Fisher, Samantha H. Cheng, Alec Christie, Steven J. Cooke, Natalie S. Dubois, Geoff Frampton, Biljana Macura, Susan J. Nichols, Rob Richards, Rebecca J. Aicher, Sara Mason, Erik Anderson, Erin Betley, Mark Borsuk, Jonah Busch, Sara Carlson, Jean-Jacques B. Dubois, Jacqualyn Eales, Edward T. Game, Robyn L. Irvine, Matthew Muir, Lydia Olander, Amina Pollard, Ana Porzecanski, Elizabeth Radke, Nicola Randall, Trevor Riley, Stephanie Ritchie, Nick Salafsky, Amanda Sigouin, Kara Stevens, Caroline E. Ridley

**Affiliations:** 1 Department of Infrastructure Engineering, The University of Melbourne, Melbourne, Victoria, Australia; 2 U.S. Environmental Protection Agency, Washington, District of Columbia, USA; 3 School of Biological Sciences, Monash University, Clayton, Victoria, Australia; 4 The Pew Charitable Trusts, Washington, District of Columbia, USA; 5 Global Science, World Wildlife Fund, Washington, District of Columbia, USA; 6 Centre for Environmental Policy, Imperial College, London, UK; 7 Canadian Centre for Evidence-Informed Conservation, Department of Biology, Carleton University, Ottawa, Ontario, Canada; 8 Environmental Incentives, South Lake Tahoe, California, USA; 9 Southampton Health Technology Assessments Centre (SHTAC), University of Southampton, Southampton, UK; 10 Stockholm Environment Institute, Stockholm, Sweden; 11 Centre for Applied Water Science, Institute for Applied Ecology, University of Canberra, Canberra, Australia; 12 Evidentiary, Darling South, Victoria, Australia; 13 American Association for the Advancement of Science, Washington, District of Columbia, USA; 14 Nicholas Institute for Energy, Environment and Sustainability, Duke University, Durham, North Carolina, USA; 15 Center for Biodiversity and Conservation, American Museum of Natural History, New York, New York, USA; 16 Center on Risk, Department of Civil and Environmental Engineering, Duke University, Durham, North Carolina, USA; 17 Conservation International, Crystal City, Virginia, USA; 18 Center for Natural Environment, U.S. Agency for International Development, Washington, District of Columbia, USA; 19 U.S. Environmental Protection Agency, Research Triangle Park, North Carolina, USA; 20 Independent Evidence Synthesis Consultant, UK; 21 The Nature Conservancy, South Brisbane, Australia; 22 Protected Areas and Establishment Conservation Directorate, Parks Canada Agency, Gatineau, Quebec, Canada; 23 International Affairs, U.S. Fish and Wildlife Service, Washington, District of Columbia, USA; 24 Centre for Evidence-Based Agriculture, Harper Adams University, Newport, UK; 25 National Oceanic and Atmospheric Administration, Oceanic and Atmospheric Research, Office of Science Support, Central Library, Silver Spring, Maryland, USA; 26 U.S. Department of Agriculture, National Agricultural Library, Beltsville, Maryland, USA; 27 Foundations of Success, Bethesda, Maryland, USA; 28 Walton Family Foundation, Washington, District of Columbia, USA

**Keywords:** assessment, conservation, ecology, environmental management, evidence, synthesis, systematic review

## Abstract

Evidence assessment—identifying, evaluating, and synthesizing data and findings from previous studies—is important to inform environmental decision-making but can be slow and resource intensive. Users seeking efficiency have developed multiple definitions and methods for rapid evidence assessment (REA), raising concerns about consistency and rigor. To improve consistency and confidence in REA, we convened an international group of evidence users and researchers to define REA for environmental applications. Through a consensus-driven and iterative approach, we define REA as: *a structured review process that aims to maximize rigor and objectivity given assessment needs and resource constraints; is transparent about trade-offs, risks, and biases; and can integrate multiple types of evidence*. Our standardized definition of REA will improve transparency and facilitate decisions about the appropriate levels of rigor required for those who commission, conduct, and use REAs for environmental decision-making.

## 1 | Introduction

Evidence assessment—identifying, evaluating, and synthesizing data and findings from previous studies—is a critical part of informed environmental management and policy decision-making (Pullin et al. 2020; CEE 2022). Evidence assessment methods are wide-ranging and include evidence synthesis methods such as systematic reviews and maps. However, systematic review, the “gold standard” for rigorous evidence synthesis, can be slow and expensive (Collins et al. 2015; Haddaway and Westgate 2019), making it impractical for many decision-making scenarios. For a variety of reasons, including the time and effort required for rigorous evidence synthesis (Pullin et al. 2004), many environmental management and policy decisions are made without considering scientific evidence in an adequate, structured, and transparent manner (Cook et al. 2010). This is a serious failing for evidence-based environmental policy and management.

In response, evidence synthesizers and users seeking quicker approaches have developed more rapid evidence synthesis approaches that go beyond “literature scans” and other rapid but non-rigorous approaches (e.g., Khangura et al. 2012; Sutherland and Wordley 2018; Garritty et al. 2021; CEE 2022). These methods go by various names, including rapid reviews (Bryant and Gray 2006), rapid evidence syntheses (Webb et al. 2017), quick scoping reviews (Collins et al. 2015), and others (see list in Grant and Booth 2009), which we collectively refer to as rapid evidence assessment (REA). These methods exist across diverse fields, including human health and environmental sciences (e.g., see Collins et al. 2015; Tricco et al. 2015; Speckemeier et al. 2022; Hamel et al. 2021). The use of REA can provide a substantial increase in access to and use of evidence to inform management decisions in many cases.

Ideally, REA methods reduce the impact of biases on evidence interpretation and provide transparent and defensible evidence to decision-makers on appropriate management- and policy-relevant timescales (e.g., weeks to months). However, an agreed-upon, formal definition of REA does not exist (Tricco et al. 2015), and there is no standard guidance for decision-makers to consider which types of assessments (including REA) best meet their needs. REA methods have often been developed for specific, discrete applications that lack rigor. This results in varied and sometimes conflicting definitions and methods with a lack of underpinning, standardized principles (Harper et al. 2024). Moreover, the great majority of these definitions have been developed for the health sciences. Tricco et al. (2015) found 30 different definitions of “rapid review” and 50 different methods in the health sciences field, with only 16 methods used more than once. These definitions are generally so loose, they are better thought of as “descriptions” that set the scope for their reviews. For example, Tricco et al. (2015) define rapid reviews as “*a type of knowledge synthesis in which components of the systematic review process are simplified or omitted to produce information in a shorter period of time*.” Almost any evidence synthesis could be argued to be consistent with this description. This lack of specificity makes it difficult for evidence synthesizers and users to make informed decisions about whether REA methods are appropriate to meet their needs and which specific REA methods would be best. In addition, confidence in the results of REAs—which can be a concern (e.g., Thomas-Walters et al. 2021)—can be increased by standardized methodological approaches and clarification that REA is different from informal, non-rigorous approaches. As an analogy, systematic review has a widely agreed definition. Anyone picking up a study that claims the title Systematic Review can have reasonable expectations that the study has followed rigorous methods consistent with that definition. If it has not, that will quickly become apparent.

To address the confusion caused by the diversity of REA descriptions and methods and the fact that they are mostly focused on health science applications, we convened an interdisciplinary group of experts to create a standard definition for environmentally focused REA and discuss its implications for potential end users. By “environmentally focused” we mean assessments related to species and ecosystem conservation; natural resource management; water, air, and soil quality; and agricultural practices. This diverse, international expert group included individuals who commission, conduct, or use evidence synthesis to address a broad range of environmental questions. Our aim was to develop a definition of REA that could help inform policy development and decision-making within the unique application space of environmental conservation and management. Here, we provide the resulting definition of REA for environmental applications and detail the rigorous process used to develop it. We also discuss key considerations that can help environmental decision-makers select REA approaches that meet their management needs to improve evidence-based decision-making.

## 2 | Methods

From late 2021 to early 2022, 39 experts in environmental evidence assessment participated in a series of five workshops on Rapid Evidence Assessment Methods and Applications (REAMA). This group was formed following a series of public webinars on REA and advertising for self-nomination through various channels, including the Collaboration for Environmental Evidence (https://environmentalevidence.org/). The size of membership was restricted because the original project aimed for an in-person workshop, and to preserve opportunities for all participants to make meaningful contributions. Participants included evidence synthesizers, users, and brokers (i.e., those who serve as knowledge bridges between synthesizers and users) associated with government agencies, nongovernmental organizations, and academic institutions from the United States, Canada, the United Kingdom, Sweden, and Australia (see Supporting Information Appendix 1). All participants worked on issues related to environmental science and management that, collectively, covered a range of specific topics, management options, and decision contexts. Although participants were mostly from the global north, there were representatives from international organizations working across developing countries and cognizant of the challenges of evidence synthesis in such environments.

The REAMA workshop series involved five half-day sessions conducted online via Zoom, with extensive use of the online workspace Mural (https://mural.co; see Supporting Information Appendix 2). All sessions had facilitators, and efforts were made to create a collegial atmosphere and encourage open sharing of ideas. Session 1 covered the motivation for developing a consensus-driven, shared definition of REA to help determine when REA could meet decision-making needs. This was followed by a survey on the critical concepts to include in a definition for REA. Survey results were used to draft an initial working definition of REA for discussion in Session 2, which was then refined over subsequent workshop sessions. During Session 3, participants identified parts of the definition to address in small groups, eliminating parts of the definition that were unnecessary, and collaboratively editing the proposed language of the working definition for feedback from all participants.

The seven-member REAMA planning committee (see Supporting Information Appendix 1) considered comments provided by participants throughout workshop discussions, editing suggestions from the small group discussions, and polling results to draft iterations of the working definition. They also developed structured activities to help resolve outstanding differences through discussion with all participants in Sessions 4 and 5. These discussions provided participants with multiple opportunities to critically appraise and contribute to the working definition as it was iteratively revised. Differences of opinion among participants were resolved where possible; where consensus was not possible, differing perspectives were documented (see Supporting Information Appendix 3). At the conclusion of the REAMA workshop series and follow-up activities, we developed a concise, standardized definition of REA supported by all workshop participants, as well as more detailed language expanding on critical concepts in that definition.

## 3 | Definition and Critical Concepts

On the basis of our deliberations, we define REA as follows:

Rapid Evidence Assessment is a structured review process that aims to maximize rigor and objectivity given assessment needs and resource constraints; is transparent about trade-offs, risks and biases; and can integrate multiple types of evidence.

This definition provides specificity lacking in earlier descriptions (e.g., Tricco et al. 2015; Hamel et al. 2021), but its brevity means that it cannot be operationalized without greater detail. Therefore, the “full” definition emerging from our process is

REA is a structured review process that aims to maximize rigor and objectivity given assessment needs and resource constraints (e.g., time). REA aims to address requirements for timely and cost-efficient decision-making while maintaining confidence in conclusions. Drawing from a variety of evidence sources, REA is typically more rigorous than less formalized practices such as traditional narrative literature reviews, but effort is reduced relative to comprehensive evidence assessment approaches such as systematic review. REA is transparent, well-documented, and the details of the specific methods used at each step are justified. Those who commission, conduct, and use REAs should be cognizant of the achievable levels of confidence in the conclusions that accompany the rapid application of different steps in the REA process.

Five interrelated critical concepts, identified by workshop participants, are included in this definition: structure, decision context, trade-offs and risks, bias, and types and sources of evidence. These concepts provide important extra detail for implementation, giving standardized guidance for how the REA definition can be used in practice. Here we explain each concept and highlight how each is explicitly included in the full definition.

### 3.1 | Structure—“REA Is a Structured Review Process”

Like systematic review, REA is a structured, standardized, multi-step process:

1. Planning/Formulating the assessment question;
2. Searching for potential evidence;
3. Selecting relevant types and sources of evidence;
4. Extracting information/data;
5. Evaluating evidence quality/validity;
6. Synthesizing evidence;
7. Drawing and communicating conclusions.

REA provides flexibility in how these methodological steps are implemented but requires clear documentation of implementation decisions at each step, the reasoning behind those decisions, and consideration of the potential consequences and limitations. Given this flexibility, planning and formulating the assessment is critical.

### 3.2 | Decision Context—“REA Aims … for Timely and Cost-Efficient Decision-Making”

Decision context refers to how evidence assessments (including confidence or uncertainty in their conclusions) will be used to inform decisions. REA is best suited for urgent or acute evidence needs, when a more comprehensive assessment would be too slow but an informal assessment would not provide enough confidence in assessment conclusions (Table 1; see Section 3.3). REA also works well to inform adaptive management decisions.

### 3.3 | Trade-Offs and Risks—“REA … Aims to Maximize Rigor and Objectivity Given Assessment Needs and Resource Constraints”

**FIGURE 1 |:**
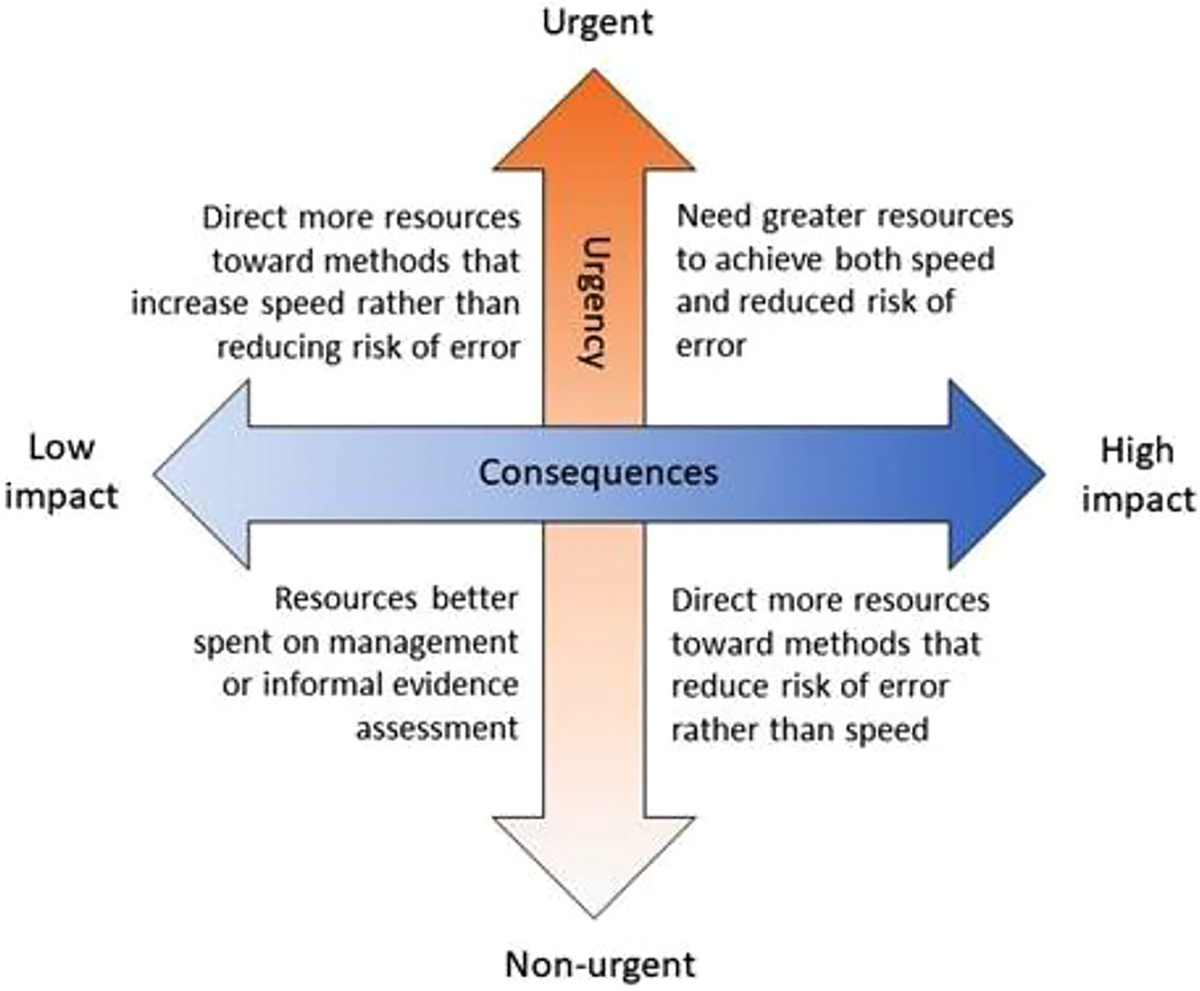
Ways in which the decision context influences choices about how to deploy resources across the stages of a rapid evidence assessment.

REA involves trade-offs relating to (1) whether to undertake REA (vs. another assessment type) and (2) the specific REA methods selected. These trade-offs often involve balancing robustness and confidence in the conclusions with speed and efficiency of the methods used throughout, and should be driven by the decision context (Figure 1). Consideration of these trade-offs and risks is crucial before undertaking REA. REA attempts to balance the different sources of risk, as it is not possible to minimize all risks simultaneously.

### 3.4 | Bias—“Those Who Commission, Conduct, and Use REAs Should Be Cognizant of the Achievable Levels of Confidence in the Conclusions”

To provide users with an appropriate level of confidence in both the assessment process and its conclusions, REA must be “bias aware.” Bias assessment is essential to build trust in the completed REA and should be transparently documented and explained to the end user. REA acknowledges potential biases stemming from (1) the types and sources of evidence used, (2) the design and validity of individual studies included, (3) the limitations of the collective evidence base, (4) the specific synthesis approaches used, and (5) the REA conclusions.

### 3.5 | Types and Sources of Evidence—“Drawing From a Variety of Evidence Sources”

REA accommodates a wide variety of evidence types and sources, including qualitative and quantitative data from diverse study types and experimental designs (Game et al. 2018). The scope of relevant evidence types and sources should be discussed as part of planning and formulating the assessment (Step 1). Decisions about the evidence to include or exclude are one way of introducing bias (Haddaway and Bayliss 2015; Nuñez and Amano 2021) and should be clearly documented and explained in the REA.

## 4 | Putting the Definition Into Practice

To illustrate how our standardized definition and related critical concepts can be applied within a case study, we examine a past REA in terms of its compliance with the new framework.

Miller et al. (2013) assessed the evidence regarding whether environmental flow management in regulated rivers could be used to reduce the encroachment of terrestrial vegetation into river channels. Although the article title erroneously refers to itself as a systematic review, the study actually used the previously published Eco Evidence method for REA (Norris et al. 2012). This method has been used in several case studies (e.g., Dahanayake et al. 2024; Wilkes et al. 2018), making it one of the more commonly used methods for environmental REA. Comparing Miller et al. (2013) to our critical concepts, Table 2 shows how our standardized definition could improve future assessments.

The comparison demonstrates that although the Miller et al. (2013) REA fares well in terms of several critical concepts, the availability of our standardized definition at the time of this research could have led to improvements. First, it is unlikely the authors would have called the study a systematic review; although it uses a systematic process, it does not follow published systematic review guidelines (CEE 2022). Second, the definition would have helped the authors to better consider the decision context and trade-offs and risks inherent in the study. Environmental water was already a prominent topic in Australia and internationally (Arthington et al. 2010) at the time this work was being done. Better consideration of the decision context and risks associated with the review conclusions may have resulted in specific recommendations for the decision context of environmental water management in Southeastern Australia.

## 5 | Conclusions and Next Steps

Given the inherent urgency in many environmental management decisions (McDonald-Madden et al. 2008), decision-makers often work with short time frames and limited resources. REA is an important tool for environmental and conservation policy development in particular; we believe our definition can help ensure that evidence assessments can happen fast enough to be useful, while also ensuring that results are rigorous enough to inform the right decision. Although REA is often framed as being “less than” assessments such as systematic review, it represents a “more than” approach to how evidence is often incorporated into environmental decisions. Moreover, we contend that a well-conducted and documented REA can be more rigorous, and thus more useful to decision-makers, than a poorly conducted systematic review. Our REA definition is intended to help decision-makers consider the level of rigor appropriate for evidence synthesis given their decision context and needs and then select appropriate methods (whether REA or a more or less rigorous option). This definition should also help commissioners and conductors of evidence assessments understand trade-offs and determine what methods best meet their needs.

The balance between rigor, speed, and other resource constraints should be determined by the decision-making context and the need for evidence (Fisher et al. 2020), including tolerance for risk and decision-maker preferences. REA is particularly useful when a relatively rigorous assessment is needed to avoid making a significant error, but available decision time frames or resources do not allow for a full gold standard systematic review. Ideally, REA allows decisions to be made with “just enough” rigor and expenditure of time and resources. Our definition of REA upholds and acknowledges the value of rapidly assessing evidence in environmental decision contexts, while clearly recognizing how trade-offs, risks, and biases affect certainty and confidence in those results.

A consistent definition for REA also helps us to understand and document how specific REA methods affect assessment conclusions, particularly as REA becomes more widely used. Comparing processes and outcomes of different forms of REA and other assessment approaches should be a focus of future research. Increased collaboration and communication among evidence generators, synthesizers, users, and brokers, particularly from the global south and other developing countries, could also facilitate the development and use of REAMA.

We acknowledge the need for future work on REA to be more inclusive of management perspectives from a wider array of countries, thereby ensuring the relevance of REA to the global environmental community. The definition presented here is not intended to be the last word on REA, but rather to provide a useful definition that facilitates uptake and trust and increases the application of REA to inform environmental decision-making—which in turn will help practitioners and decision-makers refine what REA is and how it is applied. The newly formed REAMA Community of Practice (https://reamacop.wordpress.com/) offers a collaborative space open to interested users, including particularly those users who may not have previously engaged with the literature on evidence assessment. The Community of Practice provides an opportunity for these groups to share the challenges, needs, and opportunities related to training, applications, and innovations in using REA to address environmental questions.

## Supplementary Material

Supplement1

Supporting Information

Additional supporting information can be found online in the Supporting Information section.

**Supplementary Appendix**: conl70005-sup-0001-SuppMat.docx

## Figures and Tables

**TABLE 1 |:** Examples of factors to consider when determining whether a rapid evidence assessment is the most appropriate assessment method given the decision context.

Factor to consider		Relative importance	

Impact of wrong conclusion	Low	Moderate	Moderate-to-high
Impact of delayed conclusion	High	Moderate	Low-to-moderate
Resources available	Low	Moderate	High
Tolerance for risk	High	Low-to-moderate	Low
Sources/Types of evidence to be assessed	Variable	Broad (including unpublished/unreviewed)	Narrow (primarily published/reviewed)
**Appropriate evidence assessment method**	**Quick or informal review**	**Rapid evidence assessment**	**Systematic review**

*Note*: Relative importance represents a continuum but is shown here as discrete categories to illustrate general principles.

**TABLE 2 |:** Assessment of Miller et al. (2013) study against the five critical concepts for rapid evidence assessment (REA) identified above.

Critical concept	How the concept is addressed by Miller et al. (2013)	Consideration

Structure	The Eco Evidence approach is highly structured and covers each of the steps outlined above, albeit to different levels of quality and rigor	Medium
Decision context	The study is an academic exercise, without any immediate decision context. Although the authors describe how the results could be used by managers, the study was not directly commissioned by managers seeking to make decisions about environmental water delivery	Low
Trade-offs and risks	The rigor of evidence assessment required (and the resulting risks) is not addressed in the study, other than mentioning the prohibitive workload of systematic review in general terms. This is not used as a specific reason to use Eco Evidence to undertake this assessment	Low
Bias	The study goes to considerable lengths to include a demonstrably unbiased evidence base in terms of the studies included (see Section 3.5). The Eco Evidence method uses only a superficial method to guard against bias in individual studies, giving greater weight to studies with stronger sampling designs; this is much less rigorous than formal critical appraisal of studies (Frampton et al. 2022). However, Eco Evidence may have greater capacity to include evidence contrary to a research hypothesis. It does not require summary statistics necessary to calculate standardized effect sizes in a meta-analysis; such statistics are often not provided for non-significant results (Greet et al. 2011). This feature should reduce risk of publication bias	High
Types and sources of evidence	The study was restricted to peer-reviewed journal papers accessible through the Web of Science database in an explicit effort to collect a non-biased data set (although we note the WoS does not index all journals). The article includes supplementary material detailing every reference considered for the study and explanations as to why they were or were not taken forward to the next stage of analysis	High

*Note*: The table details how each concept was incorporated into the study, with “consideration” being our subjective rating of how well the concept has been addressed.

## Data Availability

No empirical data were generated in the development of this work.
