# Supplementary material for: A Standardized Definition of Rapid Evidence Assessment for Environmental Applications
Source: Conserv Lett. Author manuscript; Available in PMC 2026 Mar 11. (PMC12973286; doi:10.1111/con4.70005)
Supplement: Supplement1 [file NIHMS2143291-supplement-Supplement1.docx]

**Supplemental Material**

Appendix 1: Workshop planning committee and participants

| **Planning Committee Members** | |
| --- | --- |
| Rebecca Aicher | American Association for the Advancement of Science, *United States* |
| Samantha Cheng | Center for Biodiversity and Conservation, American Museum of Natural History, *United States* |
| Natalie Dubois | Environmental Incentives, *United States* |
| Sara Mason | Duke University, Bridge Collaborative, *United States* |
| Caroline Ridley | U.S. EPA, *United States* |
| Kate Schofield | U.S. EPA, *United States* |
| Angus Webb | University of Melbourne, *Australia* |
| **Participants** | |
| Erik Anderson | Environmental Incentives, *United States* |
| Erin Betley | American Museum of Natural History, *United States* |
| Mark Borsuk | Center on Risk, Department of Civil and Environmental Engineering, Duke University, *United States* |
| Jonah Busch | Conservation International, *United States* |
| Sara Carlson | United States Agency for International Development, *United States* |
| Alec Christie | Centre for Environmental Policy, Imperial College, *United Kingdom* |
| Carly Cook | Monash University, *Australia* |
| Steven Cooke | Carleton University, Canadian Centre for Evidence-Informed Conservation, *Canada* |
| Jean-Jacques Dubois | U.S. EPA, *United States* |
| Jacqui Eales | Independent Evidence Synthesis Consultant, *United Kingdom* |
| Jonathan R.B. Fisher | The Pew Charitable Trusts, *United States* |
| Geoff Frampton | Southampton Health Technology Assessment Centre (SHTAC), University of Southampton, *United Kingdom* |
| Edward Game | The Nature Conservancy, *United States* |
| Robyn Irvine | Parks Canada, *Canada* |
| Biljana Macura | Stockholm Environment Institute, *Sweden* |
| Madeleine McKinnon | Bright Impact and Hillspire, *United States* |
| Matt Muir | United States Department of Interior, Fish and Wildlife Service, *United States* |
| Susan J. Nichols | University of Canberra, *Australia* |
| Lydia Olander | Nicholas Institute for Environmental Policy Solutions, Duke University, *United States* |
| Amina Pollard | U.S. EPA, *United States* |
| Ana Porzecanski | Center for Biodiversity and Conservation, American Museum of Natural History, *United States* |
| **Participants** | |
| Kent Prior | Parks Canada, *Canada* |
| Elizabeth Radke | U.S. EPA, *United States* |
| Nicola Randall | Harper Adams University, *United Kingdom* |
| Kyle Rearick | United States Agency for International Development, *United States* |
| Trevor Riley | National Oceanic and Atmospheric Administration, Oceanic and Atmospheric Research, Office of Science Support, Central Library, Silver Spring, *United States* |
| Stephanie Ritchie | United States Department of Agriculture (USDA) Agriculture Network Information Center (AgNIC) Systematic Review Working Group, *United States* |
| Rob Richards | Evidentiary, *Australia* |
| Nick Salafsky | Foundations for Success, *United States* |
| Amanda Sigouin | Center for Biodiversity and Conservation, American Museum of Natural History, *United States* |
| Sarah Solomon | American Association for the Advancement of Science, *United States* |
| Kara Stevens | Walton Family Foundation, *United States* |
| **Contractor Support** | |
| Sam Whately, Madison Lee, Parisa Shirzadi, Leah West, Megan Rooney, Kim Osborn | ICF |

Appendix 2: Workshop structure

The definition put forth in this paper was developed over the course of five virtual workshops held between November 2021 and March 2022 (in addition to some follow-on activities to clarify and confirm workshop discussions and outputs). The topical structure of each workshop is presented below. In each workshop, there was a focus on community building and open discussion, with frequent ice-breakers, gallery walks to allow participants to view and comment on the Mural boards, breakout group discussions, and report-outs from the breakout groups. Each workshop started with a recap of what was discussed and decided on in the previous workshop, and ended with a discussion of next steps. More detailed descriptions of the five workshops, including the Mural board outputs from each workshop, can be found in the *Rapid Evidence Assessment Methods and Applications to Address Environmental Questions, Workshop Series Final Summary Report* (<https://reamacop.wordpress.com/see-our-work/>).

**Workshop 1: Introduction to Rapid Evidence Assessment**

Objectives

1. Identify the types of questions, problems, and/or needs to which Rapid Evidence Assessments (REA) are currently being applied or to which users would like to apply REA.
2. Utilize examples to start shaping a common understanding of what REA is and is not.
3. Build the case for how workshop conversations and potential workshop products arising from them can result in achieving our collective desired outcomes.

Topics

1. Using examples to help describe what REA is and is not

*Breakout Group Discussions*

- *What types of questions, problems, and/or needs are REAs currently being used for? Why are they being used? How could REA be helpful for addressing existing needs but isn’t currently being applied?*
- *Develop 1-2 examples to illustrate.*
- *What can we learn from these examples to start building common understanding of what REA is and is not?*

1. Getting to Our Desired Outcomes

*Breakout Group Discussions*

- *How can we reach our collective desired outcomes with these workshops?*
- *What steps take us from workshop conversations to workshop products, to desired outcomes?*

**Workshop 2: Evidence users and synthesizers**

Objectives

1. Draft parts of a working definition of Rapid Evidence Assessment (REA).
2. Identify REA stakeholders and their roles and responsibilities.
3. Identify how different stakeholders define success when engaging in the stages of REA.

Topics

1. REA definition

*Breakout Group Discussions*

- *Trade-offs, types of evidence*
- *Structure, bias*
- *Risk, type of question being informed*

1. Actors in the evidence ecosystem
   1. Stakeholders and their roles
   2. Who are the actors in scoping, conducting, and applying REAs?
   3. What are their roles and responsibilities throughout the process?
   4. What are the problems and needs requiring evidence for which REA is clearly appropriate, clearly inappropriate, or somewhere in the middle from a user perspective?
   5. Stakeholders and defining success
2. Are certain actors and/or engagement processes more critical at some REA stages than others?
3. What does success look like from the perspective of different actors at each stage?

*Breakout Group Discussions*

- *Building value/demand for REAs*
- *Deciding to use REA/identifying the question*
- *Conducting/communicating REAs*

**Workshop 3: REA methods**

Objectives

1. Gather feedback on working definition of REA.
2. Consider utility of existing resources for understanding and communicating why and how REA should be conducted.
3. Establish collection of assessment methods that are relevant for conduct of REA, given our working definition, and explore what is gained and lost (i.e., trade-offs with each.

Topics

1. REA definition
   1. Evaluate REA working definition, including necessity of each element and language.
   2. Discuss and propose potential refinements.

*Breakout Group Discussions*

- *Structure*
- *Risk*
- *Trade-offs*

1. REA methods
   1. What assessment methods are relevant for conducting REA, given our working definition and what is gained and lost with each?

*Breakout Group Discussions*

- *Methods for searching for and selecting evidence*
- *Methods for extracting and evaluating study quality/validity of evidence*
- *Methods for synthesizing evidence and drawing/communicating conclusions*

**Workshop 4: Tools, technology, and skill sets**

Objectives

1. Gather feedback on working definition of REA.
2. Identify essential questions for a decision tree to help commissioners, synthesizers, and users agree on an appropriate evidence assessment approach to meet their requirements.
3. Draft decision trees using the essential questions.
4. Suggest existing (or missing) tools, technologies, or skill sets that can help commissioners, synthesizers, and users resolve uncertainty or disagreements about node choices on the decision trees.

Topics

1. REA definition
   1. Revised working definition of REA – is it getting better?
   2. Discuss and propose additional refinements

*Breakout Group Discussions*

- *Bias*
- *Types of questions*
- *Types of evidence*

1. REA tools, technology, and skill sets
   1. What questions are essential in a decision tree to select an evidence assessment approach?
   2. What should the tree/matrix look like? What is the starting point?
   3. What tools, technology, and skill sets exist to help navigate the tree/matrix?

*Breakout Group Discussions*

- *Design a decision tree/matrix*
- *How do you envision your tree/matrix will be used? Who is it for? How will it help distinguish between “no assessment,” “less rigorous than REA,” “REA,”, and “more rigorous than REA”?*

**Workshop 5: Putting It All Together**

Objectives

1. Achieve support for the definition of rapid evidence assessment v.0.1 and plan for definition v1.0.
2. Document achievements of the workshop series to date, who we need to reach, and how.
3. Discuss some of our outstanding commitments and how we finish the work that we started.
4. Select modes and venues for continued collaboration.

Topics

1. Reaching REA definition v.0.1

*Breakout Group Discussions*

- *Shorten “trade-offs” section?*
- *Add “decision context” section?*
- *Limit to hypothesis/claim testing or expand to other types of questions?*

1. What we have achieved so far and what we still need

*Breakout Group Discussions*

- *Decision tree/matrix/conversation guide*
- *Dashboard/menu of rapid methods*
- *Community of practice*

Appendix 3: Definitional points with less consensus

The definition of Rapid Evidence Assessment (REA) put forth in this paper resulted from the consensus-driven process detailed above (see Appendix 2: Workshop Structure). However, certain critical concepts in the definition resulted in more debate and discussion than others, highlighting that despite reaching consensus, there were several areas in which participants had differing viewpoints. For each, we erred on the side of a more broad and inclusive definition. These areas included:

- Framing REA relative to ad hoc evidence assessment and systematic review: One area of considerable discussion was whether REA always needed to be framed relative to systematic review, rather than framed as one method of evidence assessment among many possibilities, including more subjective and less transparent methods (e.g., best professional judgment). Exactly where REA sits on the gradient between less rigorous and more rigorous approaches was the subject of many discussions, with some participants expressing the view that REA, as we define it, is not an optimal, ‘just right’ sized method but rather falls closer to the ‘gold standard’ of systematic review. Some participants stressed that for many environmental management decisions, methods less rigorous than REA (i.e., ‘aluminum standard’ methods, compared to the ‘silver standard’ methods of REA) may be most appropriate—particularly in areas where no or minimal consideration of evidence is the norm.
- Sources of evidence: Another area of discussion was whether evidence considered in REA had to be from the peer-reviewed, published literature. Some participants supported the idea that REA should be based primarily (if not entirely) on peer-reviewed, published studies, given the importance of study quality in evaluating the body of evidence. Other participants supported the idea that all sources of evidence, even those more anecdotal or subjective in nature, should be considered, provided that the biases and uncertainties these types of evidence might introduce were acknowledged.
- Evaluation of study quality/risk of bias: Another area with differing viewpoints was how thorough a consideration of study quality and risk of bias REA requires. Some participants preferred a perspective in which quality appraisal was a core requirement of the REA process, while others felt that how study quality/risk of bias was considered was more context dependent.
